# Individuals adapt how they punish social norm violations through social observation

**DOI:** 10.3389/fpsyg.2025.1668877

**Published:** 2026-01-16

**Authors:** Élise Désilets, Benoit Brisson, Aude Cossette-Toutant, Karolanne Balleux, Sébastien Hétu

**Affiliations:** 1 Department of Psychology, Université du Québec à Trois-Rivières, Trois-Rivières, QC, Canada; 2 Department of Psychology, Université de Montréal, Montréal, QC, Canada

**Keywords:** adaptation, confrontation, gossip, metanorms, punishment, social exclusion, social norms

## Abstract

**Introduction:**

Metanorms are informal rules about how to react to social norm violations. Since metanorms vary across groups, individuals must adapt their metanorms to match their local social environment’s expectations. However, little is known about the mechanisms through which individuals learn and update their metanorms. The present study sought to investigate if individuals can use social observation, here observing the punitive behaviors of others, to adapt their metanorms.

**Methods:**

In an online task, 314 Canadian participants were asked to select a reaction (inaction, gossip, exclusion or confrontation) to a set of social norm violations before and after observing others who mostly used one type of punishment when faced with new social norm violations.

**Results:**

The results suggest that individuals use social observation to adapt their metanorms. Indeed, participants increased their use of the punishment they observed after observing others. This adaptation was characterized by a generalization effect suggesting that metanorm adaptation operates at an abstract level to identify the general patterns of reactions within a given social environment.

**Discussion:**

These findings provide initial evidence that metanorms can be shaped through social learning, opening new research directions to explore how this process varies across cultures, demographic groups and real-world social contexts.

## 1 Introduction

Social norms are informal rules that shape human behaviors and expectations in all societies (Bicchieri, 2005). They function better if they are enforced, especially with punitive responses in reaction to social norm violations (Fehr and Gächter, 2002). Informal rules about how to enforce social norms violations are called metanorms (Horne, 2001). Metanorms vary substantially across societies (Eriksson et al., 2017), which suggests that individuals are able to learn the metanorms of their society and adapt their enforcement behaviors in new social environments. Despite the importance of this adaptive capacity for social functioning, the learning mechanisms enabling metanorm adaptation remain unknown. Social learning could be a powerful tool to learn metanorms. With this research, we propose an original method to study adaptation of metanorms through social learning. Specifically, we explore whether observing others’ punitive responses to certain norm violations allows individuals to extract abstract enforcement rules that generalize to novel violations. Understanding how metanorms are shaped could help explain how individuals learn to efficiently enforce social norms in different social environments.

### 1.1 Literature review

Punishment is widely studied as an effective means to enforce and maintain social norms (Balafoutas et al., 2014; Carpenter et al., 2004; Fehr and Gächter, 2002; Henrich et al., 2006; Molho et al., 2020; Rudert et al., 2020; Xiao, 2018). A classification of punishment used by individuals in their daily life has recently been proposed. It suggests that individuals mostly use three kinds of punishment when faced with social norm violations: gossip, social exclusion, and confrontation (Eriksson et al., 2021; Molho et al., 2020).

Gossip consists in sharing evaluative comments (here negative comments) about an individual that is not included in the conversation (Foster, 2004). It has been shown that using gossip as punishment will establish approval or disapproval of certain behaviors, affect the reputation of the violator and contribute to maintain cooperation within a group (Shank et al., 2019). Social exclusion manifests as the deliberate and voluntary avoidance of an individual (Williams, 2006). When using gossip and social exclusion, individuals avoid direct interaction with the violator. Such responses represent a more subtle way to enforce social norms and are associated with lower social exposure and a reduced risk of retaliation (Molho et al., 2020). Hence, gossip and social exclusion are considered forms of indirect punishment (Molho et al., 2020). Confrontation consists in interacting verbally or physically with the social norm violator (Newell and Stutman, 2012). It has the advantage of having a spontaneous effect on the violator but exposed the person who produced the punishment to social disapproval, dislike or avoidance (Hildebrand et al., 2024). Confrontation is considered as a form of direct punishment (Molho et al., 2020). Alongside these punitive behaviors, individuals can also opt not to enforce the social norm (i.e., inaction). Hence, every time an individual witnesses a social norm violation, they must decide if they will enforce this norm and, if so, how. To guide their decisions, they can use metanorms (Eriksson et al., 2021).

Metanorms define how appropriate it is to punish and what type of punishment should be used. These shared expectations within a social group protect social harmony by reducing the probability of costly conflicts that can arise when punishment is improperly used (Kim, 2020; Nikiforakis and Engelmann, 2011). Indeed, metanorm violators are at risk of experiencing social costs, such as disapproval from individuals who witness the violation (Eriksson et al., 2017). It is therefore crucial for individuals to be aware and follow the metanorms of their social environment. Moreover, individuals who punish norm violators are judged as more trustworthy (Barclay, 2006; Jordan et al., 2016). Therefore, there are multiple social advantages to understanding and following metanorms of the social environment.

As proposed by Molho et al. (2020), three types of punishment are used in everyday life. This suggest that adapting metanorms is more complex than what was initially studied in terms of social norm violations punishment (i.e., punish or not punish). Indeed, following metanorms requires a certain degree of adaptability since the appropriateness to punish and the appropriateness of each type of punishment have been shown to vary across societies (Eriksson et al., 2017; Eriksson et al., 2021; Garfield et al., 2023). For instance, individuals in Canada and the United States generally find inaction more appropriate than punishment in response to a social norm violation (Eriksson et al., 2021). In contrast, 26 of the 57 countries measured by Eriksson et al. (2021) report verbal confrontation as the more appropriate response to social norm violations. Metanorms also appear capable of evolving within cultures. Cross-cultural evidence suggests that punishment systems vary systematically with socio-economic conditions—societies with food storage and economic intensification tend to use material punishments, while more egalitarian societies rely more on reputational sanctions (Garfield et al., 2023). This pattern suggests that as societies undergo socio-economic transitions, their punishment practices adapt accordingly. For such cultural-level shifts to occur, individuals within these societies must update their metanorms, either through independent adaptation to new circumstances or by observing and learning from others’ punishment behaviors. However, to our knowledge, no research has directly investigated how individuals acquire or adjust their metanorms through social observation within their own social environment.

### 1.2 Present study

We propose that the process that leads to social learning could be involved in how individuals adapt their metanorms to their social surrounding. According to Bandura’s (1969) theory of social learning, individuals learn from observing the behaviors of others. Since our everyday interactions are characterized by social norm violations that are highly diverse, individuals would need to identify more abstract rules to know how to react to them. Hence, social learning of metanorms should operate at an abstract level to help individuals understand the general pattern of reactions to social norm violations. If this is the case, individuals should be able to infer the metanorms of a local environment from observing the enforcing behaviors of others toward certain instances of social norm violations and adjust their own metanorms accordingly. Such learning through social observation would enable individuals to react to potentially any social norm violations in accordance with the social expectations, even to violations they have not yet witnessed. Previous work has shown that it is possible to change participants’ punishment behaviors to the fairness norm after observing someone else’s preference (FeldmanHall et al., 2018). However, there is no research to our knowledge that investigated if social observation could also be used to learn general enforcement behaviors that can be applied to a wide range of social norms—metanorms.

The present study sought to better understand if individuals can adapt their metanorms through the observation of others and apply them to other norm violations. To do so, we assessed if the reactions toward social norm violations can change after observing the reactions of others. Specifically, we tested if the probability of choosing different types of punishment (gossip, exclusion and confrontation) toward a set of norm violations is influenced by observing others that preferentially use a specific type of punishment (e.g., gossip) to enforce a different set of norm violations. As multiple past studies showed that individuals can change their behavior through observation in other settings (Klucharev et al., 2009; Knoll et al., 2015), we hypothesized that the participants would increase the use of the specific type of punishment favored by the others they were exposed to in the present study (e.g., the odds of choosing to use gossip will increase after observing others that preferentially used gossip).

It has been shown that the perceived inappropriateness of a behavior varies positively with the appropriateness to use punishment as an enforcement behavior (Eriksson et al., 2021). Given this correlational relationship, it is possible to hypothesize that changing how individuals react to norm violations could impact how they evaluate the inappropriateness of these violations. We thus explored if the evaluation of social norm violations can be modified by exposing participants to a social group that uses more punishment. It was hypothesized that participants would be more severe in their evaluation of social norm violations following the observation of others who use more punishment independently of which punishment is favored by the social surrounding they were presented with. All hypotheses and analyses were preregistered (https://aspredicted.org/w8tp-q97n.pdf).

## 2 Method

### 2.1 Participants

We recruited 347 individuals on the social network Facebook to complete our experiment between February 14 and March 15, 2023. The survey was advertised through the local university page. Paid advertising was used to reach a wider audience than just the individuals who follow this page. To participate in the study, individuals had to be between 18 and 65 years old and live in the province of Quebec, Canada. Thirty-three participants were excluded either because they did not answer an attention check question correctly (*n* = 32) or because they found the questions very difficult (*n* = 1; see material). The final sample includes 314 participants (*M* = 37.51 years old, SD = 12.22 years old). The participants were in majority identifying as woman (*n* = 293), white (96,5%), well educated (*M* = 18.08 years of study, SD = 3.34 years) and 36% considered themselves financially comfortable. By completing the study, participants were given the opportunity to subscribe to a draw for gift cards. This study was approved by the Comité d’éthique de la recherche – Psychologie et Psychoéducation (CERPPE; Université du Québec à Trois-Rivières; #CERPPE-22-09-07.13). Before participating, all participants gave their electronic consent.

### 2.2 Design

Participants completed a social norm violations task designed to assess evaluations of, and reactions to, social norm violations. The experiment followed a mixed design, with one within-subject factor (Block: baseline vs. post-observation; Blocks 1 and 3) and one between-subject factor (Condition: exposure to different metanorms). Blocks 1 and 3 were assessed to measure baseline and post-exposure evaluation and reaction to social norm violations. Block 2 served as the experimental manipulation, during which participants observed reactions of anonymous others, with feedback predominantly emphasizing one type of punitive reaction (gossip, exclusion, or confrontation) depending on the experimental condition. Importantly, Blocks 1 and 3 included the same set of scenarios, whereas Block 2 presented novel scenarios to prevent direct imitation of the manipulation.

### 2.3 Procedure

After reading and completing the consent forms, participants began the experiment by completing a sociodemographic questionnaire. At the end of the sociodemographic questionnaire, participants had to complete an attention check question to ensure their focus on the experiment. They then completed the three blocks of the social norm violations task. Participants concluded the experiment by answering a question about the difficulty of the task and reading a debriefing consent form informing them that the answers presented in the second block were in fact manipulated by the experimenters. All participants maintained their consent. The questionnaire and the task were presented online on the LimeSurvey platform.

### 2.4 Material

Before starting the main task, participants completed a socio-demographic questionnaire to help us characterize our sample. The questions concerned, among other things, age, sex, gender, occupation and ethnic origin.

**Figure 1:**
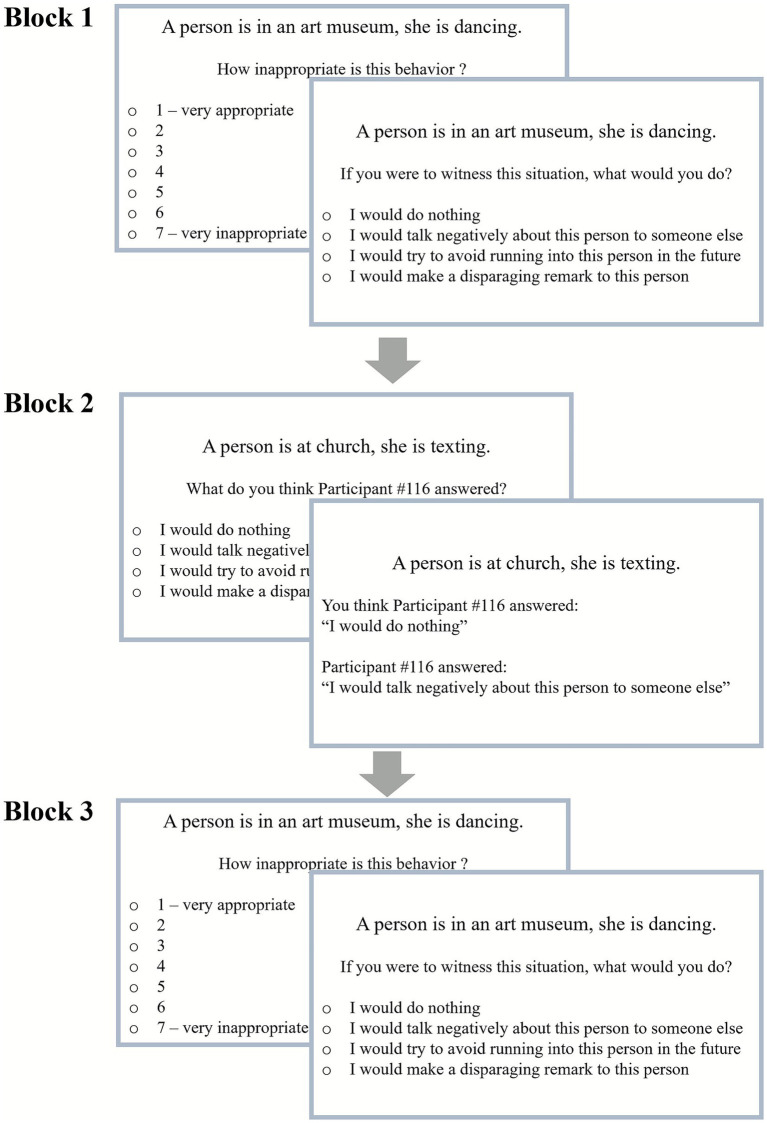
Structure of the social norm violations task across three blocks.

In the social norm violations task, inspired by Mu et al. (2015), participants saw various scenarios describing, from a third-person perspective, a situation in which a person’s behavior violates a social norm. In the actual experiment, the task was in French. Translated scenarios were taken from a previous experiment about sensitivity to social norm violations (Désilets et al., 2020). All scenarios presented a social context followed by a behavior that violates a social norm related to this social context (e.g., A person is at her children’s daycare, he/she is swearing). In the current task, 39 different scenarios were presented to participants in three blocks (Figure 1).

For each participant, a sub-set of 17 scenarios from the bank of 39 were randomly selected to be presented in the first block. The scenarios’ order of presentation was randomized. Participants were instructed that, on each trial, they would first need to rate how inappropriate they found the presented scenarios on a scale from 1 (very appropriate) to 7 (very inappropriate) and then identify which reaction, among those proposed, they would choose to do if they witnessed this situation. The second was a forced-choice question with four alternatives reactions (one non-punitive and 3 punitive choices): (a) I would do nothing (answer representing inaction), (b) I would talk negatively about this person to someone else (answer representing gossip), (c) I would try to avoid crossing this person in the future (answer representing social exclusion) and (d) I would make a derogatory remark to this person (answer representing verbal confrontation). These specific choices were inspired by research on metanorms in different societies (Eriksson et al., 2021). To familiarized participants with the task, an example was presented during the instructions.

The second block was the study’s manipulation. Scenarios used in the second block were the remaining 22 scenarios from the bank of 39: therefore, scenarios in this block were all new and participants saw these scenarios only once in the experiment. Participants were given new instructions informing them that for the next set of scenarios, they would be paired on each trial with a new anonymous individual (only identified through their ID number) who had already participated to the study. No other information about the individual was given to the participants. They were further instructed that on each trial they would see a scenario and then have to guess what reaction would the paired participant chose if they were to witness this situation (i.e., guess their answer to the question “If you were to witness this situation, what would you do?”; see Figure 1, Block 2). The same four choices of reactions as in Blocks 1 and 3 were used. Participants were also informed that after their guess, the anonymous participant’s true answer would be presented to them as feedback.

Unbeknown to the participants, the reactions of the anonymous participants were controlled by the experimenters and participants were randomly assigned to one of the three experimental conditions: gossip, exclusion, or confrontation. The feedback received from anonymous participants were previously selected to fit with the manipulation conditions in which each participant was assigned. The three experimental conditions were designed to create three different social environments with metanorms emphasizing the use of a specific punishment. Specifically, of the 22 different anonymous participants’ answers observed in Block 2, participants were exposed to 15 answers (68%) that fitted with the punishment type of the experimental condition they were in (e.g., a participant in the gossip condition would see that gossip was chosen for 15 of the 22 scenarios); four answers (18%) presenting one of the two other types of punishment (2 of each punishment type, e.g., a participant in the gossip condition would see that both exclusion and confrontation were each chosen for 2 of the 22 scenarios); and three answers (14%) where the anonymous participants would choose to do nothing. Answers were presented in the same pseudo-random order for each participant (see Supplementary Table S1). Importantly, the feedback in Block 2 was not 100% homogeneous. This design was chosen intentionally to approximate a more ecologically valid social environment, where individuals do not always react uniformly. Cross-cultural research supports this variability in punitive behavior (Eriksson et al., 2017).

In order to avoid the use of deception, responses from anonymous participants were selected from a pilot study where participants were asked how they would react if they witnessed the 39 scenarios (*n* = 58, *M*_age_ = 30.50 year, SD = 13.47 years, 79% woman). For each scenario, we found a pilot participant who answered the desired reaction and used this pilot participants ID and response as feedback. Therefore, participants were exposed to a possible social environment where real individuals would have answered to the punishment shown. Overall, this methodology allowed us to present participants with real information about anonymous participants including their true ID number and their actual answer while still being able to manipulate the type of reaction seen by our participants.

The third block was aimed at assessing participants’ evaluation of inappropriateness and reactions to social norm violations following social observation. In Block 3, participants saw in a random order the same set of 17 scenarios they previously saw in Block 1. Instructions were the same as in Block 1 (evaluation and reaction).

### 2.5 Analyses

The hypotheses, method and analyses were preregistered prior to data collection (PDF send through the submission system). All study materials are publicly available to https://borealisdata.ca/dataset.xhtml?persistentId=doi:10.5683/SP3/Z8YLDR.

First, we assessed the baseline pattern of reactions in our sample using descriptive statistics. These statistics provides a global portrait of their baseline metanorms: how our participants generally react to various social norm violations.

In line with our main objective, to test if reactions to social norm violations change after observing the reactions of others (social observation), a mixed-effects multinomial logistic regression model was used. The reaction chosen for each scenario was the dependent variable. The fixed effect independent variables were the block of scenarios (Block 1 and 3: Block 1 as reference), the inappropriateness rating (grand-mean centered), the experimental condition (gossip, exclusion, or confrontation), the interaction between inappropriateness rating and the block of scenarios and the interaction effect between the block of scenarios and the experimental condition. To control for the intra-subject variability, there was a random intercept of participants’ ID and a random slope for the Block. More details about this analysis are presented in the Supplementary material.

To explore if the evaluation of social norm violations is modified following the observation of others who use more punishment, a mixed-model linear regression was performed. The evaluation of each scenario was the dependent variable. The fixed effect independent variables were the block of scenarios (1 and 3: Block 1 as reference), the experimental condition (gossip, exclusion, or confrontation; gossip condition as reference) and the interaction effect between the Block and the condition. To control for the intra-subject variability, there was a random intercept for participants’ ID.

To control for potentially confounding variables, multiple models with sociodemographic variables (age and occupation) were tested for both regression analyses. The best models were chosen based on the Bayesian information criterion (BIC). The best models, those presented in this paper, are the models without the sociodemographic variables. Analyses were performed using R version 4.2.2 and the package lme4 (Bates et al., 2015).

## 3 Results

In this section, descriptive statistics are present, followed by the main analysis about shifts in the reaction choices to social norm violations. Finally, the change in evaluation is explored.

### 3.1 Descriptive statistics

Table 1 presents a sociodemographic description according to assigned experimental condition. Participants from each experimental condition did not significantly differ on these variables.

**Table 1:** Sociodemographic information (*n* = 314).

Condition	*n*	Age (years) Mean (SD)	Sex (% of woman)	Occupation		
				% of student	% in employment	% other
Gossip condition	102	36.98(11.96)	96.08	30.39	50.00	19.61
Exclusion condition	110	36.95 (12.73)	90.00	30.91	47.27	21.82
Confrontation condition	102	38.66 (11.78)	94.12	21.57	50.98	27.45

ANOVAs and Chi-squared confirmed that all three groups were equivalent for the presented variables, *p*s > 0.17.

**Figure 2:**
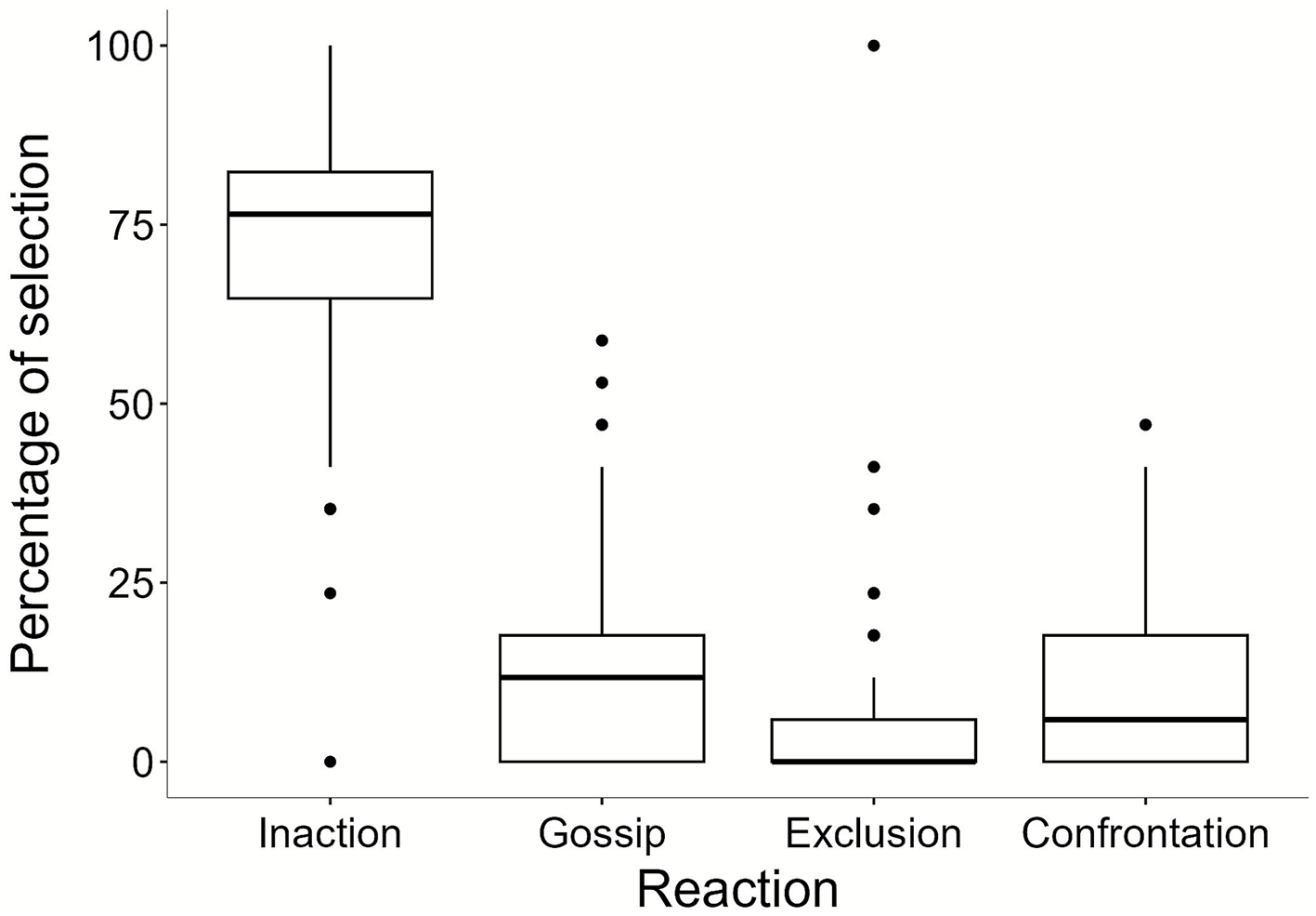
Overall baseline response pattern (metanorm) in Block 1.

In order to assess the general pattern of metanorms in our sample, we ran descriptive statistics on reactions chosen in Block 1 (Table 2). Figure 2 presents the general participants’ response pattern. Participants strongly preferred inaction in reaction to social norm violations.

**Table 2:** Punishment response rates at baseline for each experimental condition (*n* = 314).

Condition	Use of do nothing (%)		Use of gossip (%)		Use of exclusion (%)		Use of confrontation (%)	
	*M* (SD)	Median	*M* (SD)	Median	*M* (SD)	Median	*M* (SD)	Median
Gossip condition	74.68 (15.35)	76.47	12.40 (12.85)	11.76	3.69 (5.96)	0	9.23 (10.19)	5.88
Exclusion condition	69.63 (16.63)	70.59	12.41 (12.61)	5.88	6.90 (12.29)	2.94	11.07 (10.20)	5.88
Confrontation condition	75.26 (15.52)	76.47	11.53 (11.79)	5.88	3.57 (5.80)	0	9.63 (10.72)	5.88
Total	73.10 (16.02)		12.12 (12.40)		4.78 (8.80)		10.00 (10.37)	

As shown in the mixed model regression presented in the text, the three groups are equivalent in the use of gossip and confrontation, but the exclusion group is significantly higher in the use of exclusion in the first block.

### 3.2 Reaction to social norm violations

As pre-registered, a multinomial logistic mixed effect regression was performed to test for changes in the choice of punishment to social norm violations after observing choices made by others. All the results from the regression are shown in Table 3.

**Table 3:** Multinomial logistic mixed-effects model predicting punishment response choice (*n* = 314).

Predicted punishment’s response	Predictors	Estimate	Standard error	*Z* value
Gossip	Intercept	−3.18	0.22	−14.41***
	Block 3	0.94	0.18	5.34***
	Exclusion condition	0.29	0.28	1.04
	Confrontation condition	0.03	0.28	0.10
	Perception	1.33	0.06	23.22***
	Block 3: perception	0.14	0.08	1.77
	Block 3: Exclusion condition	−0.61	0.19	−3.27**
	Block 3: Confrontation condition	−0.57	0.19	−2.91**
Exclusion	Intercept	−3.76	0.26	−14.64***
	Block 3	0.89	0.24	3.68***
	Gossip condition	−0.83	0.33	−2.53*
	Confrontation condition	−0.79	0.33	−2.39*
	Perception	1.19	0.08	15.73***
	Block 3: Perception	0.21	0.11	1.95
	Block 3: Gossip condition	−0.57	0.28	−2.07*
	Block 3: Confrontation condition	−0.39	0.27	−1.45
Confrontation	Intercept	−4.03	0.24	−16.65***
	Block 3	0.58	0.24	2.36*
	Gossip condition	−0.31	0.28	−1.10
	Exclusion condition	0.27	0.27	0.99
	Perception	1.75	0.08	22.23***
	Block 3: Perception	0.25	0.11	2.17*
	Block 3: Gossip condition	−0.38	0.23	−1.62
	Block 3: Exclusion condition	−0.44	0.22	−1.97*

*p < 0.05, **p < 0.01, ***p < 0.001. Results from a multinomial logistic mixed-effects regression with random intercepts for participants. Estimates represent log-odds of selecting each punishment response compared to selecting “do nothing” (reference category). Block 1 (baseline) serves as the reference for Block 3 (post-exposure). Reference condition varies by predicted response: for Gossip, the reference is Gossip condition; for Exclusion, the reference is Exclusion condition; for Confrontation, the reference is Confrontation condition.

The model presents no differences between the conditions for the gossip and confrontation punishments in Block 1 (condition main effect) indicating that the three groups are equivalent in the use of these punishments before the manipulation. However, odds of selecting exclusion over doing nothing in the first block is lower in the gossip condition and in the confrontation condition comparatively to the exclusion condition. Although in Block 1 the exclusion group chose exclusion more often than the gossip and the confrontation group, overall, the individuals from the three conditions seem to have a very similar pattern of responses suggesting common baseline metanorms. Therefore, the difference for the exclusion group does not affect the conclusion of our main results. Indeed, since we are interested in the adaptation between Block 1 and Block 3, a slight predisposition to use exclusion does not prevent individuals from increasing their use of exclusion after being exposed to it (i.e., there is no ceiling effect).

Results show a main effect of the Block factor for all three types of punishments indicating that the odds of choosing any punishment over no punishment increase following the exposition to more punitive social environments (Block 2). In the gossip condition, the probabilities of choosing the gossip punishment increase of 3.42% from Block 1 [2.55 (1.69–3.82)] to Block 3 [5.97 (4.17–8.49)]. For the exclusion condition, the probabilities of choosing the exclusion punishment increase by 1.43% from Block 1 [1.24 (0.76–2.00)] to Block 3 [2.66 (1.74–4.07)]. Finally, for the confrontation condition, the probabilities of choosing the confrontation punishment increase by 0.64% from Block 1 [1.00 (0.63–1.58)] to Block 3 [1.64 (1.07–2.50)].

**Figure 3:**
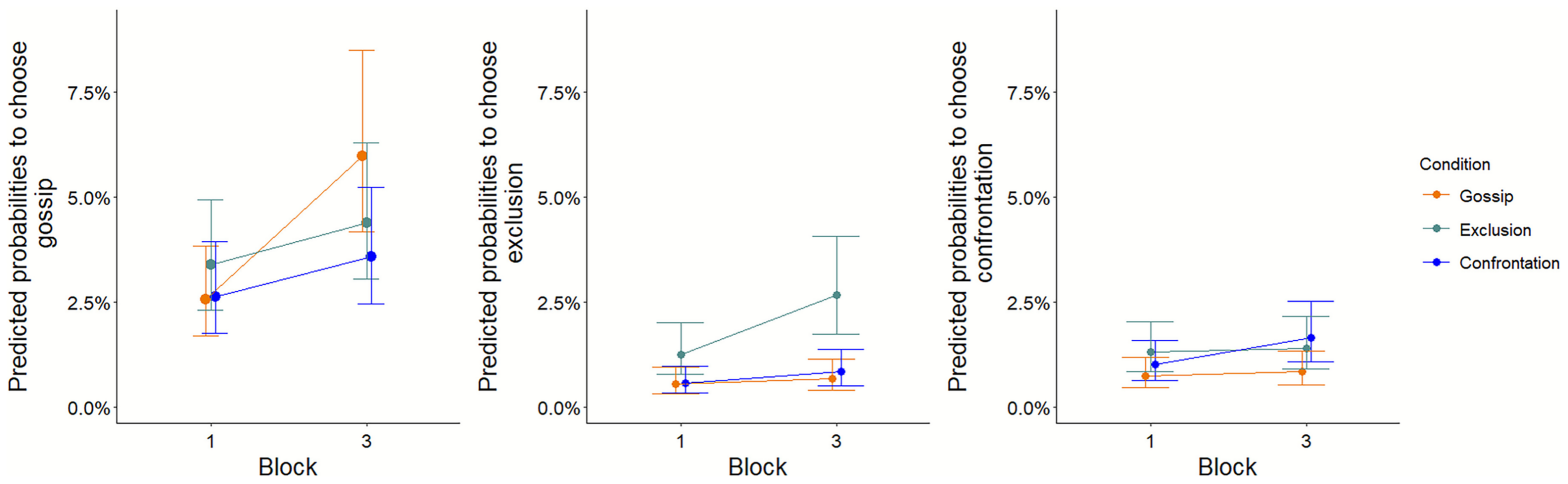
Predicted probabilities of punishment choice versus inaction across conditions. Predicted values were computed using ggeffects package in R (Lüdecke, 2018; Lüdecke, 2018).

Critically, this increase is moderated by our social learning manipulation (Figure 3) as revealed by the different Block X Condition interactions effects. Specifically, the increase in gossip selection from Block 1 to Block 3 is greater for participants in the gossip condition [who observed other anonymous participants preferentially choose gossip (+3.42%)] than for participants in the exclusion condition [who observed other anonymous participants preferentially choose exclusion (+1.01%, from 3.37 to 4.38%)] or participants in the confrontation condition [who observed other anonymous participants preferentially choose confrontation (+0.96 pp., from 2.62 to 3.58%)]. Similar, albeit less striking results, are found for the exclusion and confrontation responses. The increase from Block 1 to Block 3 in probabilities of exclusion selection is greater for participants in the exclusion condition (+1.43%, from 1.24 to 2.66%) compared to participants in the gossip condition (+0.13%, from 0.54 to 0.67%), though not significantly different from the confrontation condition (+0.27%, from 0.56 to 0.83%). The increase from Block 1 to Block 3 in confrontation selection is greater for participants in the confrontation condition (+0.64%, from 1.00 to 1.64%) compared to participants in the exclusion condition (+0.08 pp., from 1.30 to 1.38%), though not significantly different from the gossip condition (+0.09%, from 0.74 to 0.83%). Overall, these results can be seen as evidence that how participants chose to react to social norm violations is influenced by the specific punishment pattern observed. This mainly support our hypothesis, suggesting that individuals calibrated their metanorms to the social environment they were exposed to through social observation.

### 3.3 Evaluation of social norm violations

**Figure 4:**
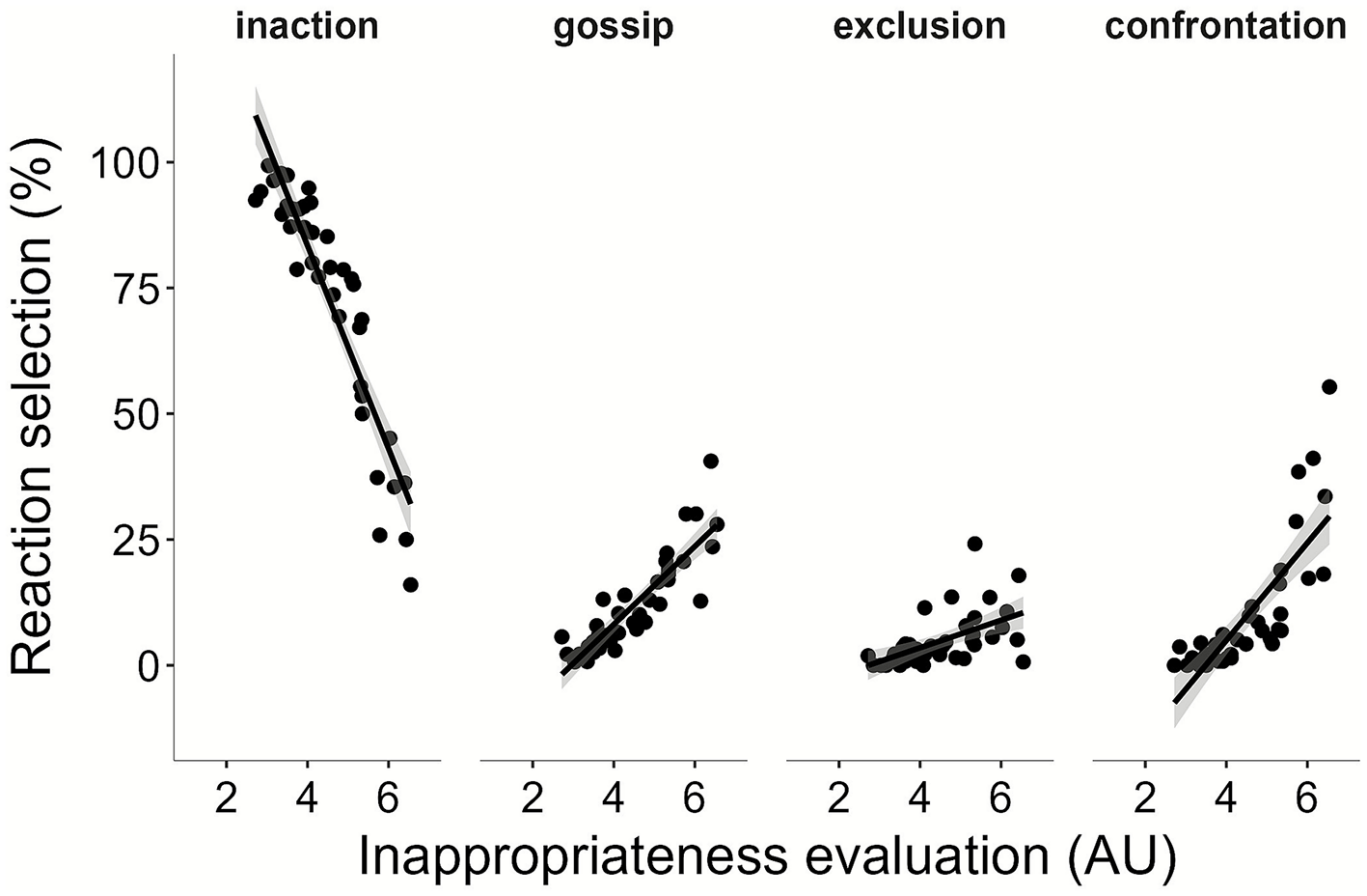
Relationship between inappropriateness evaluations and reaction selection rates at baseline (Block 1).

As a preliminary analysis, to better understand the link between how often reactions to social norm violations were chosen and how individuals evaluate the inappropriateness of these violations, correlation analyses were performed. We wanted to know if the intention to use a punishment as a reaction varies as a function of the global evaluation of inappropriateness of scenarios. To quantify the global inappropriateness evaluation for each scenario, we computed the mean of evaluation ratings in Block 1 across all participants. Concurrently, we measured the intention to use punishment by calculating the percentage of times participants chose punishment as a reaction in Block 1 for each scenario (i.e., for gossip: number of participants who chose gossip as a punishment for the scenario/number of times the scenario was seen by participants). As the variables did not meet the normality condition, Spearman Rho was used. Figure 4 presents a plot illustrating the link between the evaluation and the percentage of time each punishment was chosen. Results show a significant positive link between average evaluation of scenarios and the intention to use punishment, *rho*s (37) > 0.65, *p*s < 0.001. The more a scenario is considered as inappropriate, the higher the percentage of time a participant chose punishment as a reaction to it. The link is reversed for inaction. The less inappropriate a scenario is considered, the more often people choose inaction, *rho* (37) = −0.94, *p* > 0.001.

Each graph represents the link between the mean evaluation in Block 1 and the percentage of time the reaction was chosen by participants in Block 1 for the 39 scenarios. The higher the rating, the more inappropriate the behavior is considered to be.

To explore if individuals modified their evaluation of social norm violations between the first and the third block, a mixed effect regression model was used. Results show that neither the Block, the condition or the interaction effect between Block and condition can significantly predict the evaluation of inappropriateness of the scenarios (see Table 4). This result suggests that individuals did not change their evaluation of violation after observing other individuals use punishment more frequently.

**Table 4:** Mixed-effects model predicting inappropriateness evaluation (*n* = 314).

Predictors	Estimate	Standard error	*t* value
Intercept	4.53	0.08	57.85***
Block 3	0.06	0.05	1.08
Exclusion condition	0.04	0.11	0.33
Confrontation condition	−0.10	0.11	−0.87
Block 3: Exclusion condition	0.04	0.08	0.59
Block 3: Confrontation condition	0.03	0.08	0.45

*p < 0.05, **p < 0.01, ***p < 0.001. Results from a mixed-effects regression with random intercepts for participants. Estimates represent log-odds of selecting each punishment response compared to selecting “do nothing” (reference category). Block 1 (baseline) serves as the reference for Block 3 (post-exposure). For the condition, Gossip serve as reference.

Although there is no general effect of Block across all scenarios, it is still possible that participants increased their evaluation only for some specific scenarios. As secondary analysis, we wanted to look for potential change in evaluation, but only for scenarios for which participants switched from inaction in the first block to a punishment in the third block. In the pre-registration, this analysis was planned to be a mixed ANOVA. However, for the sake of continuity with the previous analysis and methodological rigor, a mixed-effect regression was applied. Results from the mixed-effect regression model show an increase from Block 1 to Block 3 in the evaluation of inappropriateness for these scenarios, *b* = 0.23, *p* = 0.03, but no effect of condition (*b* < −0.10, *p* > 0.13) or interaction between Block and conditions (*b* < 0.12, *p* > 0.42). Therefore, when only considering scenarios for which participants switched their reaction from doing nothing to using punishment, participants increased their evaluation of inappropriateness after observing more punitive social environments.

Overall, our second hypothesis concerning changes in evaluation is not supported. However, the secondary analysis provides some insight, suggesting that changes in reactions are associated with changes in evaluation, although these effects are not strong enough to emerge at a global level.

## 4 Discussion

Studies about social norms often describe punishment as a widely used means to enforce social norms (Fehr and Gächter, 2002; Henrich et al., 2006; Wu et al., 2016). However, some studies have shown that inaction can often be considered as more appropriate in response to everyday social norm violations (Balafoutas et al., 2014; Eriksson et al., 2021). Accordingly, societies seem to have different expectations about how one should react to social norm violations (e.g., punish or not and how to punish). These social norms about reactions to social norm violations are called metanorms (Eriksson et al., 2017; Horne, 2001). The present preregistered study sought to test whether individuals can adapt their metanorms to their local social environment by observing the reactions of others to various everyday social norm violations. Specifically, we tested if observing individuals who reacted to a set of norm violations by preferentially using a specific type of punishment could influence the choice of reactions made by our participants toward a different set of norm violations. We also explored if observing a more punitive social environment would increase the evaluation of the inappropriateness of social norm violations. We hypothesized that participants would choose more often the specific punishment used by the social environment they were exposed to and that they would be more severe in their evaluation of social norm violations after observing more punitive reactions. Our results support most of our hypotheses about the role of social observation in our ability to calibrate our metanorms to more punitive social environments.

### 4.1 Key findings

We first assessed the metanorms in our sample by characterizing the baseline pattern of reactions to various social norm violations. Participants preferred inaction instead of punishing norm violators. This finding is consistent with previous research indicating a tendency to avoid punishment in looser and more individualistic societies (Eriksson et al., 2021). Indeed, all our participants lived in Quebec, Canada, a mostly loose and individualistic culture (Suh et al., 1998; Zourrig et al., 2018). These types of cultures are associated with greater tolerance for norm deviations (loose cultures) and greater priority to personal goals versus collective’s goals (individualistic cultures; (Triandis, 1989)). Beyond the reproduction of previous studies, the observed baseline pattern highlights the fact that punishment is not always the preferred reaction to a social norm violation, at least in several western societies (FeldmanHall et al., 2018).

The findings of this research confirm the main hypothesis of this study about the role of social observation in our ability to adapt our metanorms. After observing more punitive behaviors in others, participants adapted their own choice of reactions to social norm violations by increasing their use of punishment. Previous work had already shown that social observation can change how individuals react to violations of the fairness social norm in economic tasks (FeldmanHall et al., 2018; House et al., 2020). The present study extends this role to shaping metanorms which regulate how individuals enforce the wide range of social norms characterizing their everyday lives.

### 4.2 Theoretical implications

The experimental setup allowing participants to choose between different punitive behaviors showed that individuals seem to adapt their metanorms to the social environment they are navigating. Our design, with three conditions serving as mutual controls, allowed us to test pattern-specific learning rather than general conformity effects. Indeed, observing individuals predominantly choosing one type of punishment mostly raised the likelihood of selecting this specific punishment in reaction to subsequent social norm violation. For example, the use of gossip was greater for participants who observed others mainly use gossip than for participants who observed others mainly use confrontation or exclusion. Similar, albeit smaller, effects were found for the confrontation and exclusion punishments. Participants who witnessed others use confrontation or exclusion had higher likelihood to choose the specific type of punishment they observed shortly after. However, this increase in the likelihood was only significantly higher in comparison to one of the two other experimental conditions but was also accompanied by a rise in the utilization of punitive measures in general.

The ease with which gossip was specifically adopted raises the possibility that this type of punishment may possess unique characteristics that facilitate its specific incorporation into the metanorms of our sample. Notably, among the three types of punishment, gossip emerged as the most frequently employed punishment in Block 1 of the study. Having metanorms that already favor gossip may have facilitated the learning of this type of punishment compared to other types of punishment.

While previous research has provided evidence that individuals can adapt their reactions to specific types of norms violations, such as the fairness norm (FeldmanHall et al., 2018; House et al., 2020), current results show that they can also calibrate their reactions to a wide range of social norms violations that can be encountered in daily life. Previous research used a paradigm where changes in reactions to a norm violation was measured after observing others react to the same violations (e.g., inequal splits of resources in studies on the fairness norm) suggesting a possible stimulus–response learning. We posit that everyday interactions, characterized by diverse and potentially new social norm violations, necessitate the identification of metanorms: general and abstract rules about norm enforcement. Importantly, the finding that observing others’ reactions to certain norm violations changes individuals’ reactions to other violations (i.e., generalization effect) aligns with the notion that adaptation of metanorms should transcend the confines of a stimulus–response level, where individuals merely observe others’ specific reactions to specific norm violations. Instead, metanorms adaptation appears to operate at a more abstract level, focusing on discerning the general patterns of reactions to social norm violations within a given social environment.

Although punishment is associated with the evaluation of social norm violations (Eriksson et al., 2021; Mendoza et al., 2014), participants, overall, did not change their evaluation of the inappropriateness of social norm violations despite increasing their use of punishments. However, when analyses focussed only on scenarios for which participant changed their reaction from doing nothing to a punishment, we observed an increase in their inappropriateness evaluation. This result suggests a link between the reactive and evaluative processes of norm enforcement and raises a question about the potential direction of this relation: does our choice of reaction guide our evaluation of the inappropriateness or vice-versa? Further research where both types of social information are observed in others and changes in both evaluations and reactions are measured are necessary to better understand the organization of the system supporting norm enforcement.

### 4.3 Limitations

The present study has some limitations. First, we proposed only punishment or inaction as possible reactions to social norm violations although other reactions could be preferred by participants. Indeed, FeldmanHall et al. (2018) showed that individuals may rather choose reactions in line with compensative justice over sanctions when it is possible. Therefore, it is possible that the high percentage of inaction responses hide some other, non-punitive reactions. However, our choice of reactions was based on prior work that have suggested that these particular reactions represent a realistic set that covers the behaviors associated with witnessing social norms violations in everyday life (Molho et al., 2020) and across cultures (Eriksson et al., 2021). Furthermore, since much of the previous research focused on the use of punishment to study norm enforcement, our intention was to gain a better understanding of how individuals adapt their metanorms about punishment. Now that it has been shown that individuals can learn metanorms with a task such as the one presented in this research, future research should expand the choice of reactions to social norm violations beyond punishment including less punitive reaction such as politely ask to stop the behavior or tell the violator that his behavior is considered odd or inappropriate.

Second, the characteristics of our sample limits the generalization of our findings. We conducted our research on individuals from Quebec, Canada recruited via Facebook. This sample is from a culture considered as individualistic (Suh et al., 1998), limiting our results to be generalize to collectivistic cultures. Also, since social learning can vary across cultural contexts, further reinforcing the caution with which the present findings should be generalized (Mesoudi et al., 2016). Additionally, the sample was composed of majority of white, well-educated women, which also restricts the generalizability of our findings. While we remain confident in the fundamental pattern observed (that individuals adjust their metanorms based on social observation), future research with more diverse samples is essential to determine whether the magnitude of these effects varies across demographic groups and cultural contexts.

### 4.4 Conclusion

In conclusion, the results show that observing others’ reactions to a subset of norm violations can modify the intention of individuals to use punishment to subsequent violations, even beyond the subset of violations they were exposed to. Therefore, this research highlights the potential role of social observation in how cross-cultural differences in metanorms can emerge, be maintained or modified.

## Data Availability

The datasets presented in this study can be found in online repositories. The names of the repository/repositories and accession number(s) can be found at: https://borealisdata.ca/dataset.xhtml?persistentId=doi:10.5683/SP3/Z8YLDR, Repository name: Borealis – Canadian Dataverse Repository DOI: 10.5683/SP3/Z8YLDR.
